# Severe Pulmonary Differentiation Syndrome Despite Corticosteroid Prophylaxis in Acute Promyelocytic Leukemia: Clinical Improvement With Pulse Methylprednisolone Therapy and High-Flow Nasal Cannula

**DOI:** 10.7759/cureus.113285

**Published:** 2026-07-24

**Authors:** Paul Cardozo, Lucia Reynolds, Carlos Rivera, Cinthia Berrio, Favio Hurtado

**Affiliations:** 1 Critical Care Medicine and Neurocritical Care, Clínica de Las Américas, Santa Cruz de la Sierra, BOL; 2 Critical Care Medicine, Hospital Obrero No. 3, Caja Nacional de Salud, Santa Cruz de la Sierra, BOL; 3 Hematology, Clínica de Las Américas, Santa Cruz de la Sierra, BOL; 4 Critical Care Medicine, Clínica de Las Américas, Santa Cruz de la Sierra, BOL

**Keywords:** acute promyelocytic leukemia, acute respiratory failure, all-trans retinoic acid, differentiation syndrome, high-flow nasal cannula, methylprednisolone

## Abstract

Differentiation syndrome (DS) is a potentially life-threatening complication of differentiation therapy in acute promyelocytic leukemia (APL), particularly with all-trans retinoic acid (ATRA) and arsenic trioxide. We report the case of a 37-year-old woman with newly diagnosed APL who developed severe pulmonary DS 48 hours after ATRA initiation despite dexamethasone prophylaxis. She developed progressive hyperleukocytosis, fluid retention, severe hypoxemia, and diffuse bilateral pulmonary infiltrates, requiring high-flow nasal cannula (HFNC) support with an FiO₂ of up to 80% and a flow rate of 60 L/min, combined with awake prone positioning. The initial PaO₂/FiO₂ ratio was 150, and the respiratory rate-oxygenation (ROX) index was 4, indicating a clinical profile associated with a higher risk of intubation. In the absence of bacterial growth from respiratory samples or a cardiogenic cause, DS was considered the predominant mechanism of respiratory deterioration. After 48 hours of HFNC support, the ROX index increased to 8; however, persistent pulmonary involvement prompted escalation to pulse methylprednisolone therapy at 1 g/day for three days. The patient showed clinical, oxygenation, and radiologic improvement, with an increase in the PaO₂/FiO₂ ratio from 150 to 350, and invasive mechanical ventilation was avoided. This case highlights the importance of early recognition of severe pulmonary DS in APL and suggests that individualized corticosteroid intensification combined with advanced noninvasive respiratory support may be useful in selected high-risk patients.

## Introduction

Acute promyelocytic leukemia (APL) is a biologically and clinically distinct subtype of acute myeloid leukemia defined by the promyelocytic leukemia-retinoic acid receptor alpha (PML-RARA) fusion protein, which blocks myeloid differentiation. It represents a hematologic emergency because of its high risk of early mortality, primarily related to hemorrhagic complications from simultaneous activation of coagulation and fibrinolysis [1]. In this setting, immediate initiation of all-trans retinoic acid (ATRA) upon clinical, morphologic, or cytogenetic suspicion of APL is considered a cornerstone of initial management. Early administration of ATRA plays a fundamental role in the initial treatment of APL. Its ability to induce differentiation of leukemic promyelocytes contributes to the rapid correction of disease-associated coagulopathy and reduction of early mortality. Accordingly, major international guidelines recommend initiating ATRA at the earliest clinical suspicion of APL, even before definitive molecular confirmation [1].

Although ATRA has substantially changed the natural history of APL and remains an essential component of frontline therapy, differentiation therapy may trigger differentiation syndrome (DS), a potentially fatal complication characterized by fever, dyspnea, pulmonary infiltrates, hypotension, renal dysfunction, serous effusions, and weight gain [2,3]. The reported incidence of DS ranges from 2.5% to 37%, reflecting differences in diagnostic criteria, induction protocols, and concomitant chemotherapy use [3-5]. In contemporary practice, corticosteroid prophylaxis has been incorporated for high-risk patients; however, several studies have shown that this strategy reduces DS severity but does not completely prevent its occurrence, with reported rates ranging from 11% to 29% despite steroid prophylaxis [5,6]. The persistence of DS despite corticosteroid prophylaxis may be explained by mechanisms beyond leukocyte count control, including endothelial activation, cytokine release, capillary leak, and tissue migration of differentiating blasts [7].

From a regional perspective, APL is of particular epidemiologic relevance in Latin America, where its relative frequency among acute leukemias is consistently higher than that reported in North America and Europe, and early mortality remains elevated [8,9]. DS is commonly diagnosed on clinical grounds, based on the appearance of compatible manifestations such as fever, dyspnea, pulmonary infiltrates, hypoxemia, fluid retention, serous effusions, hypotension, or renal dysfunction after initiation of differentiation therapy. Its recognition may be delayed because there is no single confirmatory laboratory or imaging test, and its clinical features frequently overlap with sepsis, severe pneumonia, pulmonary leukostasis, transfusion-related acute lung injury (TRALI), transfusion-associated circulatory overload (TACO), and acute respiratory distress syndrome (ARDS). In complex cases, diagnostic attribution is often strengthened retrospectively by clinical improvement after corticosteroid therapy, once alternative causes have been reasonably evaluated. Concomitant infections and limited early access to specialized hematologic assessment may further complicate timely recognition.

We report the case of a patient with newly diagnosed APL who developed severe pulmonary DS despite dexamethasone prophylaxis, associated with progressive hyperleukocytosis and acute respiratory failure. The patient was managed with pulse methylprednisolone, high-flow nasal cannula (HFNC), and awake prone positioning, with subsequent clinical and oxygenation improvement, and invasive mechanical ventilation was avoided. This case highlights the diagnostic challenges of severe pulmonary DS in APL and suggests that advanced noninvasive respiratory support may be considered in carefully selected patients with close monitoring, particularly when hypoxemic respiratory failure is present but immediate standard indications for invasive mechanical ventilation are absent.

## Case presentation

A 37-year-old woman had a remote history of a cesarean section complicated by chronic intermittent infection of the Pfannenstiel surgical wound, characterized by recurrent local discharge and multiple courses of empiric antibiotic therapy through self-medication, without definitive resolution. Her additional medical history included prior episodes of post-COVID-19 bronchospasm and recent odontalgia in the days preceding admission, treated with nonsteroidal anti-inflammatory drugs. The patient presented with a subacute history of general malaise, asthenia, adynamia, and fatigue associated with pain in the right lower limb. During the initial evaluation, superficial venous thrombosis of the small saphenous vein was documented in the setting of significant hematologic cytopenias. On admission, laboratory studies showed severe leukopenia of 1,100 cells/mm³, anemia, and marked thrombocytopenia of 23,000 cells/mm³, accompanied by elevated inflammatory markers. The key laboratory and oxygenation findings during the clinical course are summarized in Table 1.

**Table 1:** Key laboratory and oxygenation findings during the clinical course. HFNC, high-flow nasal cannula; ROX, respiratory rate-oxygenation

Parameter	Admission/diagnosis	Respiratory deterioration/HFNC	During HFNC and methylprednisolone	Follow-up	Reference range	Unit
White blood cell count	1,100	61,500	12,800	1,200	5,000-10,000	cells/mm³
Platelet count	23,000	31,000	42,000	35,000	150,000-400,000	cells/mm³
Absolute neutrophil count	748	30,135	1,152	240	2,500-8,000	cells/mm³
PaO₂/FiO₂ ratio	320	150	180	350	>300	ratio
ROX index	Not applicable	4	8	14	Not applicable	ratio
C-reactive protein	260	97	88	45	0-6	mg/L
Procalcitonin	1.54	0.29	0.12	0.1	<0.5	ng/mL
Fibrinogen	344	233	228	169	180-350	mg/dL
International normalized ratio	1.3	1.26	1.24	1.02	0.9-1.3	ratio
D-dimer	39	14	12	12	0-0.5	mg/L

**Figure 1:**
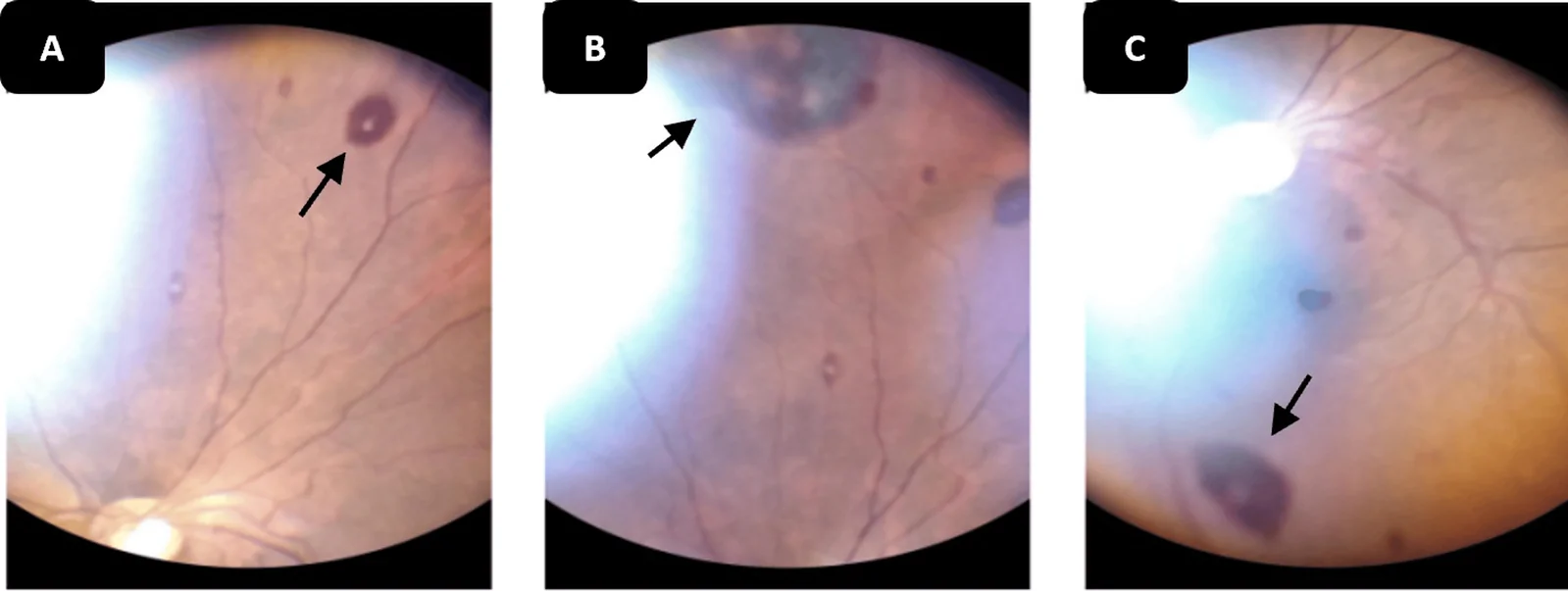
Fundoscopic examination revealing bilateral Roth spots. (A) Right eye, posterior pole, showing multiple rounded retinal hemorrhages with pale centers, consistent with Roth spots, including macular involvement. (B) Right eye, peripheral retina, showing additional hemorrhagic lesions, illustrating the multifocal distribution of the retinopathy. (C) Left eye, showing a peripheral hemorrhagic lesion with sparing of the macular region. Both right-eye images were included because they show complementary retinal regions, documenting both posterior pole involvement and peripheral extension of the hemorrhagic lesions. These findings were interpreted as hemorrhagic retinopathy secondary to the underlying hematologic disorder.

During the initial clinical course, the patient developed methicillin-sensitive *Staphylococcus aureus *bacteremia and severe pneumonia, for which targeted antimicrobial therapy was initiated. Concurrently with the hematologic workup, she experienced a rapid reversal of her leukocyte profile, progressing from severe leukopenia to marked hyperleukocytosis, with a peak white blood cell count of 61,500 cells/mm³. Diagnostic evaluation confirmed APL through detection of the PML-RARA rearrangement. After confirmation of APL, funduscopic examination revealed bilateral Roth spots, with macular involvement of the right eye (Figure 1).

Given the history of *S. aureus* bacteremia, echocardiographic evaluation was performed and showed no evidence of vegetations or other findings suggestive of infective endocarditis. Therefore, the retinal findings were interpreted primarily in the context of leukemia, anemia, and severe thrombocytopenia. Following diagnostic confirmation, differentiation therapy with ATRA was initiated at a dose of 90 mg/day according to internationally accepted treatment protocols in a setting where arsenic trioxide (ATO) was unavailable. Given the progressive hyperleukocytosis, hydroxyurea at 1,500 mg/day was added as a cytoreductive measure during the induction phase. Daunorubicin 120 mg was also administered according to the institutional protocol. In parallel, intensive, protocolized, and closely monitored transfusion support was implemented to manage consumptive coagulopathy and the high hemorrhagic risk characteristic of APL during the initial phase of the disease. In addition, dexamethasone was administered at a total daily dose of 32 mg/day as prophylaxis for DS.

**Figure 2:**
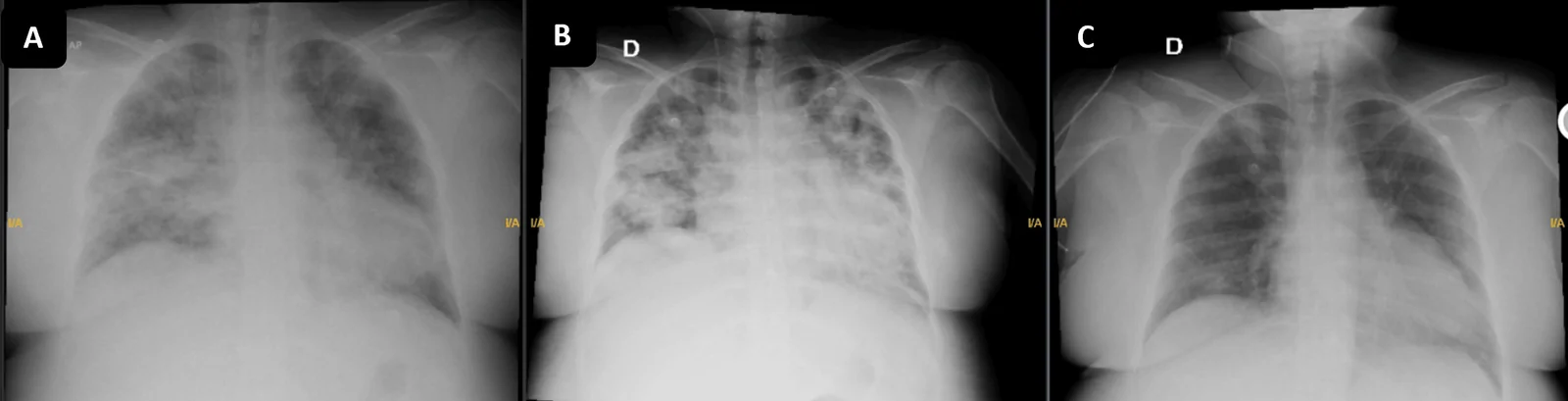
Serial chest radiographs during pulmonary DS. (A) Chest radiograph obtained 48 hours after ATRA initiation, showing diffuse bilateral alveolar-interstitial opacities, predominantly in the perihilar and basal lung fields, consistent with severe pulmonary involvement in the setting of acute hypoxemic respiratory deterioration. (B) Follow-up radiograph after 48 hours of HFNC support, showing persistent bilateral alveolar-interstitial opacities, which prompted escalation to pulse methylprednisolone therapy. (C) Follow-up radiograph obtained after three days of pulse methylprednisolone therapy and continued HFNC support, showing marked radiologic improvement with significant resolution of the bilateral pulmonary opacities. ATRA, all-trans retinoic acid; DS, differentiation syndrome; HFNC, high-flow nasal cannula

Despite steroid prophylaxis, 48 hours after initiation of ATRA, the patient developed rapidly progressive respiratory deterioration characterized by worsening hypoxemia, diffuse bilateral pulmonary infiltrates (Figure 2A), a marked increase in oxygen requirements, and fluid retention with weight gain exceeding 5 kg. At that time, the PaO₂/FiO₂ ratio was 150, compatible with moderate hypoxemic respiratory failure and a clinical profile associated with a higher risk of intubation. The clinical presentation posed a complex diagnostic challenge that included sepsis-related ARDS, severe pneumonia, pulmonary leukostasis, opportunistic viral or fungal infection, diffuse alveolar hemorrhage, TRALI, TACO, drug-induced lung injury, and cardiogenic pulmonary edema. These possibilities were evaluated according to the clinical course, microbiologic findings, hemodynamic status, transfusion history, radiographic pattern, echocardiographic findings, inflammatory biomarker trends, and response to therapy. Active infectious progression was considered less likely because respiratory samples showed no bacterial growth, and inflammatory biomarkers decreased during the respiratory course. Cardiogenic pulmonary edema and TACO were considered less likely because there was no clinical or echocardiographic evidence of cardiac failure or predominant volume overload. Diffuse alveolar hemorrhage was considered in the setting of thrombocytopenia and coagulopathy, but there was no hemoptysis or clinical evidence of active pulmonary bleeding. Overall, the close temporal relationship with ATRA initiation, marked hyperleukocytosis, fluid retention, predominant pulmonary involvement, and the absence of a more likely alternative cause supported pulmonary DS as the predominant mechanism of respiratory deterioration.

Given the progression of respiratory failure, HFNC support was initiated, reaching an FiO₂ of up to 80% and a flow rate of 60 L/min, combined with awake prone positioning. The respiratory rate-oxygenation (ROX) index was 4, suggesting a higher risk of intubation. However, invasive mechanical ventilation was not immediately pursued because the patient remained hemodynamically stable, awake, cooperative, and without altered mental status, severe ventilatory failure, or clinical signs of respiratory exhaustion. Although close monitoring was maintained because of the high risk of deterioration, the absence of immediate standard indications for intubation and the need to minimize invasive procedure-related risks in the setting of severe thrombocytopenia and coagulopathy supported a trial of HFNC. After 48 hours of HFNC support, the ROX index increased to 8, compatible with partial functional improvement, supporting the decision to continue noninvasive respiratory support under close surveillance. However, given the persistence of radiographic pulmonary involvement (Figure 2B), severe pulmonary-predominant DS despite dexamethasone prophylaxis, marked hyperleukocytosis, and concern for ongoing inflammatory lung injury related to cytokine-mediated endothelial activation and capillary leak, corticosteroid therapy was intensified using pulse methylprednisolone at a dose of 1 g/day for three days. This decision was made as an individualized rescue strategy rather than as a standard replacement for dexamethasone-based management, in the context of rapidly progressive hypoxemic respiratory failure and the need to avoid invasive mechanical ventilation if clinically feasible.

After initiation of pulse methylprednisolone therapy, the patient showed a favorable clinical and oxygenation response, with improvement in the PaO₂/FiO₂ ratio from 150 to 350, progressive reduction in oxygen requirements, and respiratory stabilization, thereby avoiding the need for invasive mechanical ventilation. Follow-up chest imaging showed significant improvement in the bilateral pulmonary infiltrates (Figure 2C), consistent with the favorable clinical course and response to pulse methylprednisolone therapy, HFNC support, and awake prone positioning. The patient subsequently experienced progressive respiratory improvement, allowing gradual withdrawal of HFNC support and transition to low-flow oxygen therapy. However, her subsequent course was complicated by profound neutropenia, with a nadir absolute neutrophil count of 240 cells/mm³, and persistent severe thrombocytopenia, with a platelet count of 35,000 cells/mm³, in the context of the underlying disease and daunorubicin-associated myelotoxicity. For this reason, she required ongoing intensive transfusion support and treatment with filgrastim 300 mcg every 24 hours. At the time of intensive care unit discharge, the patient had sustained respiratory improvement, had been weaned from HFNC support, and remained stable on low-flow oxygen therapy, with a PaO₂/FiO₂ ratio of 350 and a ROX index of 14. She was ultimately discharged from the intensive care unit for continued specialized hematologic management.

At the four-month outpatient follow-up, laboratory assessment showed hematologic recovery, with values within the normal range. Molecular monitoring of bone marrow was performed using quantitative reverse transcription-polymerase chain reaction for t(15;17) PML-RARA, showing no detectable molecular expression, with a ratio of 0.00000 and an expression rate of 0.000%. These findings were compatible with a complete molecular response and supported a favorable clinical and hematologic status at follow-up.

## Discussion

DS is one of the most relevant and potentially fatal complications associated with the treatment of APL using differentiating agents such as ATRA and ATO. Since its initial description as “retinoic acid syndrome” in 1992, it has been recognized as a systemic inflammatory condition characterized by dyspnea, pulmonary infiltrates, unexplained fever, fluid retention, serosal effusions, hypotension, and renal dysfunction, with the potential to rapidly progress to multiorgan dysfunction if not promptly treated [3]. Despite the remarkable improvement in APL outcomes achieved with differentiation therapies, with cure rates exceeding 90% in modern protocols such as the ATRA/ATO regimen reported in the landmark trial by Lo-Coco et al. published in the New England Journal of Medicine, DS remains an important cause of early morbidity and mortality during induction therapy [2].

In the present case, the patient developed severe pulmonary-predominant DS despite dexamethasone prophylaxis, with progressive respiratory deterioration occurring within days of ATRA initiation. This temporal pattern is consistent with the classic time window for DS onset, which typically occurs within the first two weeks of exposure, as described in contemporary reviews and the European LeukemiaNet recommendations [1]. Weight gain exceeding 5 kg, progressive hypoxemia, and diffuse pulmonary infiltrates were consistent with severe DS according to the PETHEMA severity scoring system [10]. The subsequent requirement for advanced respiratory support with HFNC further reflected the severity of pulmonary involvement. The respiratory manifestations observed in this case represent one of the most challenging diagnostic scenarios in the intensive care unit because of their overlap with severe pneumonia, sepsis, pulmonary leukostasis, and noncardiogenic ARDS [6,7].

As detailed in the Case Presentation, opportunistic infection, diffuse alveolar hemorrhage, TRALI, TACO, drug-induced lung injury, and cardiogenic pulmonary edema were considered, and bronchoscopy with bronchoalveolar lavage was considered but not performed because of severe hypoxemia, marked thrombocytopenia, and a high hemorrhagic risk. Nevertheless, several findings supported DS as the predominant pathophysiologic mechanism, including the close temporal relationship with ATRA initiation, progressive hyperleukocytosis with a marked peak during induction, significant fluid retention, and the absence of evidence supporting cardiogenic failure as the primary explanation for respiratory deterioration. Recent studies have confirmed that both a rapid increase in leukocyte count and peak leukocyte levels during induction are independent predictors of DS, even among patients receiving steroid prophylaxis [11,12]. The decision to initiate hydroxyurea for cytoreduction in our patient is consistent with current recommendations for the management of differentiation-associated hyperleukocytosis, given its association with severe DS and pulmonary complications [13]. Current evidence suggests that the pathophysiology of DS extends beyond the leukocyte count itself and involves a complex inflammatory process mediated by cytokine release, endothelial injury, capillary leak syndrome, and tissue migration of differentiating blasts into target organs, particularly the lungs [7,8]. Sanz and Montesinos described DS as sharing pathophysiologic features with systemic inflammatory response syndrome, driven by the release of proinflammatory mediators, increased capillary permeability, and alterations in cellular adhesion induced by ATRA [5].

These mechanisms may help explain both the fulminant pulmonary presentation observed in our patient and the rapid clinical improvement following intensive anti-inflammatory treatment. The universally accepted cornerstone of DS management is the immediate administration of high-dose dexamethasone, with temporary discontinuation of ATRA and/or ATO generally reserved for patients with severe respiratory dysfunction or severe renal impairment [1,5]. However, evidence regarding optimal treatment intensification strategies in rapidly progressive severe DS remains limited. In our case, given the imminent risk of invasive mechanical ventilation, treatment was escalated to pulse methylprednisolone therapy (1 g/day for three days), followed by significant improvement in oxygenation and radiologic findings within the first 48 hours. Although pulse methylprednisolone therapy is not included in current standard recommendations and there are no randomized clinical trials specifically evaluating its use for DS, it may be considered only as an individualized rescue strategy in selected cases of severe DS with rapidly progressive respiratory deterioration and an imminent risk of invasive mechanical ventilation [6,7].

A distinctive aspect of this case was that respiratory failure was managed with advanced noninvasive respiratory support, a particularly relevant consideration in patients with APL, in whom invasive mechanical ventilation may be associated with hemorrhagic and infectious complications in the setting of profound thrombocytopenia and coagulopathy [6,7]. HFNC has been shown to improve oxygenation and reduce respiratory workload in immunocompromised patients with acute respiratory failure, representing a useful strategy to avoid invasive mechanical ventilation in selected patients [14]. Furthermore, studies involving patients with hematologic malignancies suggest that HFNC is safe and may be associated with improved outcomes through a reduced need for endotracheal intubation [15]. However, HFNC should not delay invasive mechanical ventilation when standard indications for intubation are present. In this case, as described in the Case Presentation, continued HFNC support was considered appropriate because the patient remained clinically stable and showed improvement in the ROX index from 4 to 8 after 48 hours. In this setting, rather than immediately discontinuing ATRA, early treatment intensification with pulse methylprednisolone was prioritized and combined with HFNC and intermittent awake prone positioning. This strategy was associated with rapid and sustained clinical, oxygenation, and radiologic improvement during the critical phase of the syndrome, supporting consideration of HFNC as a context-dependent option before escalation to invasive mechanical ventilation in carefully selected patients with APL and severe thrombocytopenia or coagulopathy.

## Conclusions

This case highlights the diagnostic complexity of severe pulmonary DS in APL despite dexamethasone prophylaxis. The close temporal relationship with ATRA initiation, hyperleukocytosis, fluid retention, predominant pulmonary involvement, absence of bacterial growth from respiratory samples, and subsequent improvement after corticosteroid intensification supported DS as the predominant mechanism of respiratory deterioration. Early escalation to pulse methylprednisolone therapy, combined with HFNC support and awake prone positioning, was associated with rapid clinical, oxygenation, and radiographic improvement, avoiding invasive mechanical ventilation in a high-risk patient. This case reinforces the importance of early recognition and individualized treatment intensification in patients with severe pulmonary DS, rapidly progressive hypoxemic respiratory failure, and a high procedural risk associated with invasive mechanical ventilation because of severe thrombocytopenia or coagulopathy.
